# Understanding how cancer survivors’ needs and experiences after treatment impact their health care utilization: a survey-administrative health data linkage study.

**DOI:** 10.23889/ijpds.v7i3.1801

**Published:** 2022-08-25

**Authors:** Robin Urquhart, Cynthia Kendell, Julia Kaal, Jessica Vickery, Lynn Lethbridge

**Affiliations:** 1 Dalhousie University; 2 Nova Scotia Health

## Objectives

To link population-based survey data to routinely collected administrative health data to enable investigation of how cancer survivors' ongoing physical, emotional, and practical needs and experiences after completing cancer treatment impact their healthcare utilization, including discharge from oncology to primary care.

## Approach

The "Cancer Transitions Survey" is a population-based survey examining survivors' experiences and needs after completing cancer treatment. The survey was administered by the Nova Scotia Cancer Registry (NSCR) as part of a national study, the largest of its kind in Canada. Respondents included Nova Scotian survivors of breast, melanoma, colorectal, prostate, hematologic, and young adult cancers who were 1-3 years after treatment. Survey responses were linked to cancer registry, physicians' claims, hospitalization, and ambulatory care data. The data linkage provided a full four years of healthcare utilization data for each cancer survivor, beginning one year after their cancer diagnosis.

## Results

1557 survivors responded to the survey and subsequently had their data linked. Collectively, breast, colorectal, and prostate cancer survivors represented 78.5% of survey respondents. Most respondents (65.3%) were 65 years of age or older and 69.8% had an existing co-morbid condition. Regression analyses are now being conducted to investigate whether the type and magnitude of post-treatment care needs, and the interventions (services and supports) received, impact health care utilization in the survivorship period, including discharge to primary care.

## Conclusion

This study represents a unique opportunity to link data unavailable in administrative health data: namely, self-reported needs and use of non-physician services and supports (e.g., support groups, counselling). As such, this dataset permits investigation of healthcare utilization and patterns of care that cannot be accomplished using administrative health data alone.

